# Antibody‐positive autoimmune encephalitis and paraneoplastic neurological syndrome: A Swedish case series

**DOI:** 10.1002/brb3.3534

**Published:** 2024-05-03

**Authors:** Sonja Kosek, Joachim Burman, Anna Rostedt Punga

**Affiliations:** ^1^ Department of Medical Sciences Uppsala University Uppsala Sweden; ^2^ Centre for Clinical Research and Education Karlstad Sweden

**Keywords:** anti‐N‐methyl‐D‐aspartate receptor encephalitis, autoimmune diseases, encephalitis, paraneoplastic syndromes

## Abstract

**Objective:**

This study aimed to explore the clinical characteristics and temporal disease course of patients with autoimmune encephalitis (AE) and paraneoplastic neurological syndrome (PNS) in Sweden.

**Methods:**

Thirty‐seven antibody‐positive AE and PNS cases were identified in the Healthcare region Mid Sweden between 2015 and 2019. Clinical data were collected through a retrospective review of electronic health records. Patients were divided into three subgroups based on antibody type: neuronal surface antibodies (NSAbs), onconeural antibodies, and anti‐GAD65 antibodies.

**Results:**

Nineteen patients had NSAbs, 11 onconeural antibodies, and seven anti‐GAD65 antibodies. Anti‐LGI1 and anti‐NMDAR were the most frequently detected NSAbs, with anti‐NMDAR cases having an older‐than‐expected age distribution (median age 40, range 17–72). Only 11 of 32 (30%) of patients had findings suggesting encephalitis on initial MRI, but 28 of 31 (90%) had pathological findings on initial cerebrospinal fluid analysis. All patients but one had abnormal EEG findings. Median time to immunotherapy was comparable among the three subgroups, whereas patients with anti‐LGI1, anti‐CASPR2, and anti‐IgLON5 had an eightfold longer time to immunotherapy than anti‐NMDAR and anti‐GABA‐B (*p* = .0016). There was a seasonal variation in onset for patients with non‐tumor‐related NSAbs and anti‐GAD65 antibodies, with most patients (72%) falling ill in spring or summer.

**Conclusion:**

Swedish patients with AE and PNS had similar clinical characteristics as previously described cohorts from other geographical regions except for anti‐NMDAR encephalitis, with older onset than expected. The onset of non‐tumor‐related AE occurred predominantly in the warm seasons, and AE with a more insidious onset was associated with delayed treatment initiation.

## Graphical abstract

This case series of 37 patients with autoimmune encephalitis (AE) and paraneoplastic neurological syndromes (PNS) demonstrated that Swedish patients have similar clinical characteristics compared with previously described cohorts from other geographical regions except for anti‐NMDAR encephalitis, with older onset than expected. The onset of non‐tumor‐related AE occurs predominantly in the warm seasons, and AE with a more insidious onset is associated with delayed treatment initiation.

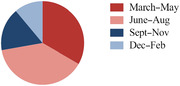

## 1 INTRODUCTION

Autoimmune encephalitis (AE) and paraneoplastic neurological syndrome (PNS) are immune‐mediated nervous system disorders. Both are associated with autoantibodies targeting neuronal antigens. These neuronal antibodies are typically divided into two groups: neuronal surface antibodies (NSAbs) targeting membrane‐bound antigens such as cell surface receptors and onconeural antibodies targeting intracellular antigens. NSAbs are considered pathogenic and can be detected in patients with both AE and PNS (Sun et al., 2020). Patients with AE associated with NSAbs often respond well to immunotherapy and early initiation of therapy is associated with a favorable prognosis (Titulaer et al., 2013). Onconeural antibodies are generally not pathogenic but may be markers of an underlying cancer. In patients with onconeural antibodies, finding and treating the underlying cancer is considered the most important (Binks et al., 2022). The diagnosis of AE and PNS relies not only on autoantibody testing but also on clinical examination and ancillary tests, such as magnetic resonance imaging (MRI), electroencephalogram (EEG), and cerebrospinal fluid (CSF) analysis (Graus et al., 2016, 2021). The diagnosis of AE and PNS can be elusive as the ancillary testing is often normal or unspecific, leading to delayed treatment and prolonged patient suffering.

Like several other autoimmune disorders, such as multiple sclerosis (Koch‐Henriksen & Sørensen, 2010), AE and PNS display some geographical variations, which have been attributed to both genetic and environmental factors (Alentorn et al., 2023; Binks et al., 2018; Mueller et al., 2018; Titulaer et al., 2013).

The purpose of this study was to present a comprehensive description of patients with AE and PNS in Healthcare region Mid Sweden based on a review of their electronic medical records to discover potential differences from previously described cohorts from other geographical areas.

## 2 METHODS

### 2.1 Study population

The study population consisted of 37 patients recruited from our previous epidemiological study of AE and PNS (Kosek et al., 2023), in which all patients in Healthcare region Mid Sweden who tested positive for a neuronal antibody between 2015 and 2019 were included. All patients who met the diagnostic criteria for definite AE according to Graus et al. (2016) or the diagnostic criteria for definite PNS according to the PNS‐Care Score proposed by Graus et al. (2021) were included in this case series. Five patients with anti‐GAD65‐associated disorders (stiff person syndrome disorder [SPSD], encephalitis, or cerebellopathy) and one with anti‐IgLON5 encephalopathy were also included. All participants resided in Healthcare region Mid Sweden, Sweden's second‐largest healthcare region with more than 2.1 million inhabitants (2020, Statistics Sweden). Healthcare region Mid Sweden is serviced by two university hospitals (Uppsala University Hospital and Örebro University Hospital), five county hospitals, and 18 rural hospitals.

### 2.2 Antibody testing

Neuronal antibodies were analyzed by one of two laboratories (Uppsala University Hospital or Wieslab AB). NSAbs were analyzed using an indirect fixed cell‐based immunofluorescence assay (Euroimmun AG), except anti‐neurexin‐3α antibodies, which were analyzed using indirect immunofluorescence. Antibodies targeting intracellular antigens (onconeural antibodies) were analyzed using a combination of indirect immunofluorescence and immunoblot or only immunoblot. Anti‐GAD65 antibodies were analyzed in one laboratory using immunoblot and indirect immunofluorescence, with quantification of serum samples using ELISA. The other laboratory used only immunoblot and quantification with ELISA. Patients were grouped into three groups according to the subtype of neuronal antibody detected: (1) NSAbs: anti‐NMDAR, anti‐LGI1, anti‐CASPR2, anti‐GABA‐B, anti‐IgLON5, anti‐AMPA 1/2, anti‐DPPX, and anti‐neurexin‐3α; (2) onconeural antibodies: anti‐Yo, anti‐Hu, anti‐Ri, anti‐Ma2/Ta, anti‐CV2, anti‐amphiphysin, anti‐SOX1, anti‐Zic4, and anti‐Tr; and (3) anti‐GAD65 antibodies. Both laboratories tested for the antibodies mentioned above except for anti‐IgLON5, anti‐DPPX, and anti‐neurexin‐3α antibodies, which were only analyzed by one of the laboratories.

### 2.3 Review of medical records

A retrospective review of all patients’ complete medical records was performed. Demographic data, information about coexisting conditions, and symptoms at presentation and during the disease course were collected. Results of the diagnostic work‐up were collected and included (a) neuronal antibody testing (serum and CSF); (b) computed tomography (CT) scans; (c) whole‐body 18‐Fluorodeoxyglucose positron emission tomography (FDG‐PET); (d) brain MRI; (e) EEG (all patients had a routine EEG with 17 or 21 electrodes); and (f) CSF analysis. The review also included information about the temporal disease course and treatment, including (a) the department where the patient was first seen; (b) time to diagnosis (days from first contact to detection of auto‐antibody); (c) time to immunotherapy (days from first contact to start of immunotherapy); (d) type of immunotherapy; (e) length of hospital and intensive care unit (ICU) stays; (f) follow‐up time (time from detection of auto‐antibody to death or last date when the patient's electronic medical records were checked); (g) relapses (defined as recurrence of symptoms after at least 3 months of improvement after initial immunotherapy, with the recurrence of symptoms requiring renewed treatment with immunotherapy); and (h) the month when the first symptom started.

All patients were included in the analysis of seasonal variation, with an exception for those who met the criteria for definite PNS. In patients with definite PNS, we considered cancer as the trigger of disease independent of a potential seasonal trigger such as infection.

### 2.4 Statistical analysis

Categorical variables were presented as percentages and numerical variables were described as mean values with standard deviation or medians with ranges. The Mann–Whitney *U* test was used for comparisons between two groups, and the Kruskal–Wallis test was used for comparisons between multiple groups. Statistical analysis was performed using GraphPad Prism version 9.3.1 for Mac (GraphPad Software; www.graphpad.com).

### 2.5 Standard protocol approvals, registrations, and patient consent

This study was approved by the Swedish Ethical Review Authority (Dnr: 2019‐03068). Written informed consent was obtained for all patients, except for deceased patients, for whom consent was presumed (in line with the ethical application).

## 3 RESULTS

### 3.1 Clinical characteristics

**FIGURE 1:**
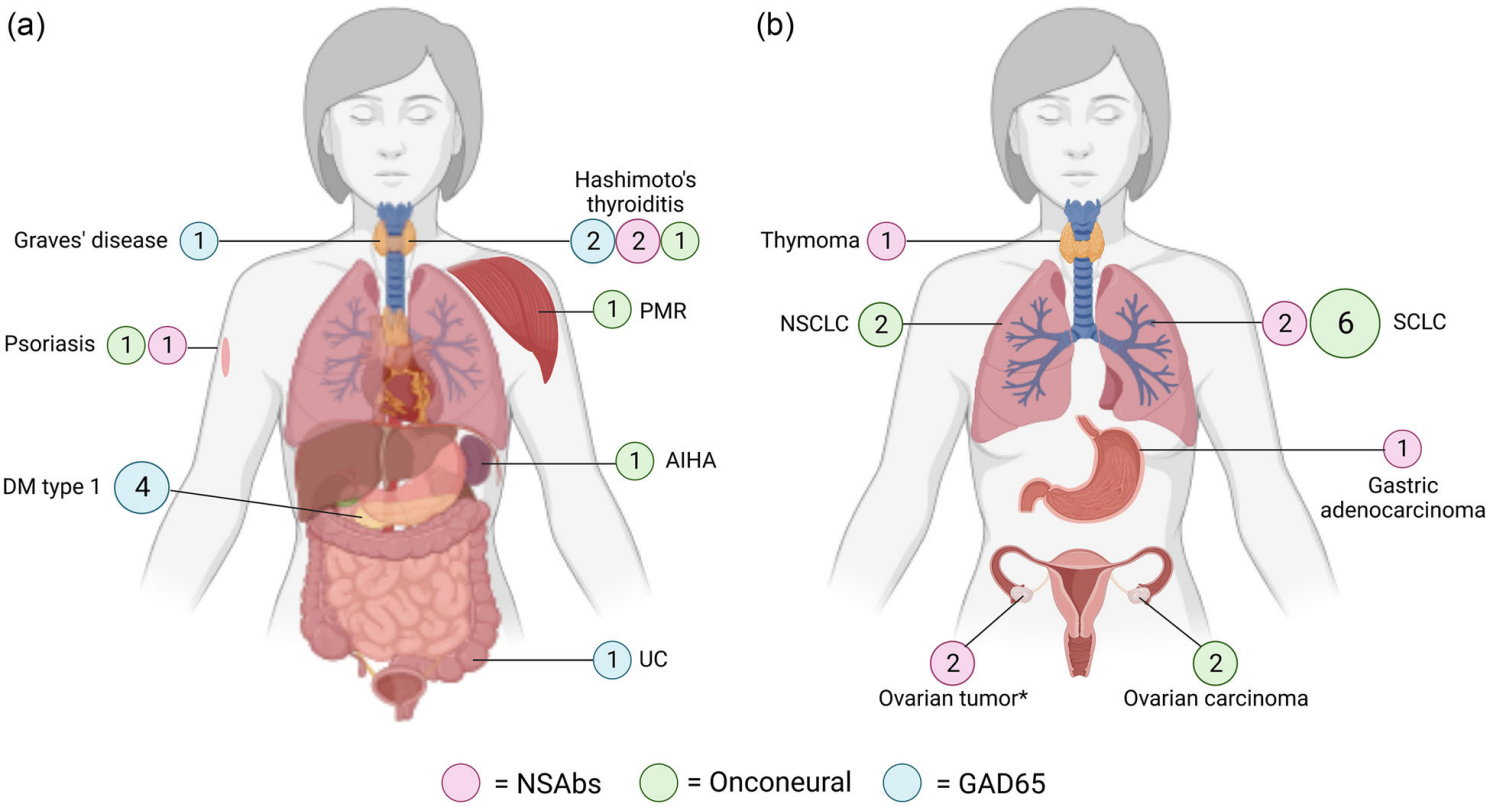
(a) Coexistent autoimmune diseases in the study population (14/37 patients). Circles are color‐coded according to antibody subtype, and numbers inside the circles indicate the number of patients diagnosed with the disease. (b) Cancer diagnosed in the study population within 2 years of disease onset (16/37 patients). Circles are color‐coded according to antibody subtype, and numbers inside the circles indicate the number of patients diagnosed with the disease. *Ovarian tumor was detected in two patients with anti‐NMDAR encephalitis, but both died before definite PAD was acquired. AIHA, autoimmune hemolytic anemia; DM, diabetes mellitus; NSAbs, neuronal surface antibodies; NSCLC, non‐small‐cell lung cancer; PMR, polymyalgia rheumatica; SCLC, small‐cell lung cancer; UC, ulcerative colitis.

Thirty‐seven patients were included in this study; 19 had NSAbs, 11 had onconeural antibodies, and seven had anti‐GAD65 antibodies (Table 1). In the NSAbs subgroup, 11 of 19 (58%) were female, and the median age was 69 (range 17–80). In the patients with onconeural antibodies subgroup, 6 of 11 (55%) were female, and the median age was 67 (range 56–83). Four of the seven patients with anti‐GAD65 antibodies were females (57%), and the median age was 62 (range 50–75). In the NSAbs subgroup, anti‐NMDAR antibodies (83% females, median age 40, range 17–72) and anti‐LGI1 antibodies (33% females, median age 75, range 61–76) were the most common. Limbic encephalitis was the most frequent clinical syndrome in all three subgroups, occurring in 89% of patients with NSAbs, 45% with onconeural antibodies, and 43% with anti‐GAD65 antibodies. Psychiatric symptoms were among the most common initial symptoms (*n* = 13, 68%) for patients with NSAbs, frequently in combination with one more symptom (most often cognitive dysfunction) (Table 2). Two patients presented with only psychiatric symptoms at their first healthcare visit to their GPs. Almost half of the patients with NSAbs had seizures at first presentation, and during the entire disease course, an additional 25% had seizures at some point. Five of six patients with anti‐LGI1 encephalitis had faciobrachial dystonic seizures (FBDS), which in all five cases was a presenting symptom. In the onconeural subgroup, a third of the patients had cerebellar ataxia at presentation and a subsequent diagnosis of paraneoplastic cerebellar degeneration. Among the five patients with onconeural antibodies who presented with limbic encephalitis, cognitive dysfunction (80%) was the most common presenting syndrome, and two (40%) had psychiatric symptoms at initial presentation. All patients with anti‐GAD65‐associated limbic encephalitis presented with seizures at their first healthcare visit. Cancer was found in 16 of 37 patients (43%), and 14 of 37 (38%) had a coexisting autoimmune disease (Figure 1).

**TABLE 1:** Age, sex, diagnostic criteria, antibody test results (including antibody titer when available), and clinical presentation for all included patients in this study. Six patients did not meet the diagnostic criteria; stiff person syndrome disorder (*n* = 2), anti‐GAD65‐associated limbic encephalitis or cerebellopathy (*n* = 3), and anti‐IgLON5 encephalopathy (*n* = 1).

Case	Age	Sex	Diagnostic criteria	Antibody serum	Antibody CSF	Clinical presentation
**1**	61	M	Definite AE	LGI‐1 (1:100)	Not analyzed	Anti‐LGI1 encephalitis
**2**	73	F	Definite AE	LGI1	Negative	Anti‐LGI1 encephalitis
**3**	75	F	Definite AE	LGI‐1 (1:10)	Not analyzed	Anti‐LGI1 encephalitis
**4**	74	M	Definite AE	LGI‐1 (1:10)	Negative	Anti‐LGI1 encephalitis
**5**	76	M	Definite AE	LGI1	Negative	Anti‐LGI1 encephalitis
**6**	75	M	Definite AE	LGI‐1 (1:100)	Not analyzed	Anti‐LGI1 encephalitis
**7**	35	F	Definite NMDAR	NMDAR	NMDAR	Anti‐NMDAR encephalitis
**8**	72	F	Definite NMDAR	Negative	NMDAR (1:16)	Anti‐NMDAR encephalitis
**9**	34	M	Definite NMDAR	Negative	NMDAR	Anti‐NMDAR encephalitis
**10**	17	F	Definite NMDAR	Negative	NMDAR (1:1)	Anti‐NMDAR encephalitis
**11**	68	F	Definite NMDAR	Not analyzed	NMDAR	Anti‐NMDAR encephalitis
**12**	45	F	Definite NMDAR	Negative	NMDAR	Anti‐NMDAR encephalitis
**13**	69	F	Not fulfilled	IgLON5 (1:1000), SOX1	IgLON5	Anti‐IgLON5 encephalopathy
**14**	78	M	Definite AE	CASPR2 (1:1000)	CASPR2	Limbic encephalitis
**15**	80	F	Definite PNS, definite AE	GABA B (1: > 10,000)	GABA B	Paraneoplastic limbic encephalitis
**16**	60	M	Definite AE	CASPR2 (1:1000)	Not analyzed	Limbic encephalitis
**17**	66	M	Definite PNS, definite AE	GABA B (1:1000)	GABA B	Paraneoplastic limbic encephalitis
**18**	53	F	Definite PNS	CASPR2 (1:1000), LGI‐1 (1:100)	Negative	Morvan's syndrome
**19**	75	F	Definite PNS, definite AE	NMDAR (1:100), AMPA (1:10), Hu (1:100, blot intensity 28), Amphiphysin (blot intensity 12)	NMDAR, Hu, CV2, amphiphysin	Limbic encephalitis
**20**	78	M	Definite AE	Ma2/Ta	Negative	Limbic encephalitis
**21**	81	M	Definite PNS, definite AE	Ma2/Ta	Not analyzed	Paraneoplastic limbic encephalitis
**22**	67	M	Definite PNS, definite AE	Hu	Hu + Zic4	Paraneoplastic limbic encephalitis
**23**	83	F	Definite PNS	Not analyzed	CV2	Paraneoplastic neuropathy
**24**	72	F	Definite PNS	Not analyzed	Hu	Paraneoplastic neuropathy
**25**	67	F	Definite PNS	Hu (blot intensity 130), Yo (blot intensity 31)	Hu	Paraneoplastic neuropathy
**26**	67	F	Definite PNS	Yo (1:10,000, blot intensity 87)	Not analyzed	Paraneoplastic cerebellar degeneration
**27**	65	M	Definite PNS	CV2 (1:100, blot intensity 17)	CV2	Paraneoplastic cerebellar degeneration
**28**	56	F	Definite PNS	Yo (1:1000, blot intensity 110)	Yo	Paraneoplastic cerebellar degeneration
**29**	77	F	Definite PNS, definite AE	Ma2/Ta	Ma2/Ta	Paraneoplastic limbic encephalitis
**30**	66	M	Definite PNS	Not analyzed	Hu	Paraneoplastic limbic encephalitis
**31**	60	F	Definite AE	GAD65 (ELISA > 2000 IU/mL, blot intensity 28)	Not analyzed	Limbic encephalitis
**32**	54	F	Not fulfilled	GAD65	GAD65	GAD65‐associated cerebellopathy
**33**	62	M	Not fulfilled	GAD65 (ELISA 150,080 IU/mL)	GAD65	Stiff person syndrome disorder
**34**	75	F	Not fulfilled	GAD65 (ELISA > 1,000,000 IU/mL, blot intensity 40)	GAD65	GAD65‐associated cerebellopathy
**35**	64	M	Not fulfilled	GAD65 (ELISA 17,639 IU/mL)	Not analyzed	Limbic encephalitis
**36**	50	M	Not fulfilled	GAD65 (ELISA 615,324 IU/mL, blot intensity 30)	GAD65	Stiff person syndrome disorder
**37**	68	F	Definite AE	GAD65 (ELISA > 1,000,000 IU/mL, blot intensity 47)	Not analyzed	Limbic encephalitis

Abbreviations: AE, autoimmune encephalitis; CSF, cerebrospinal fluid; PNS, paraneoplastic neurological syndrome.

**TABLE 2:** Presenting symptoms at first contact with the health care system and symptoms at any stage of the disease for each antibody subgroup.

	Presenting symptoms			Symptoms at any stage		
	NSAbs, *n* (%)	Onconeural, *n* (%)	GAD65, *n* (%)	NSAbs, *n* (%)	Onconeural, *n* (%)	GAD65, *n* (%)
Seizures	9 (47)	2 (18)	3 (43)	14 (74)	3 (27)	3 (43)
FBDS^a^	5 (83) ^α^	0 (0)	0 (0)	5 (83)^a^	0 (0)	0 (0)
Psychiatric symptoms	13 (68)	2 (18)	0 (0)	16 (84)	4 (36)	2 (29)
Cognitive dysfunction	13 (68)	4 (36)	1 (14)	18 (95)	5 (45)	3 (43)
Cerebellar ataxia	0 (0)	4 (36)	2 (29)	0 (0)	4 (36)	2 (29)
Speech disturbance	3 (16)	2 (18)	1 (14)	5 (26)	2 (18)	1 (14)
Movement disorder	3 (16)	3 (27)	2 (29)	6 (32)	3 (27)	2 (29)
Decreased consciousness	1 (5)	0 (0)	0 (0)	3 (16)	2 (18)	0 (0)
Autonomic dysfunction	1 (5)	0 (0)	0 (0)	6 (32)	1 (9)	0 (0)
Neuropathy	0 (0)	1 (9)	2 (29)	0 (0)	4 (36)	0 (0)
Hyponatremia	3 (16)	1 (9)	0 (0)	5 (26)	2 (18)	0 (0)

*Note*: Patients can be represented in more than one row if they had a combination of symptoms. Movement disorders include gait abnormalities, stereotypic movements, fasciculations, muscle cramps and stiffness, myokymia, myoclonus, and myorhythmia.

Abbreviations: FBDS, faciobrachial dystonic seizures; NSAbs, neuronal surface antibodies.

^a^ Calculated on all cases of anti‐LGI1 encephalitis, *n* = 6.

### 3.2 Diagnostic workup

#### 3.2.1 Neuronal antibodies

Neuronal antibodies were analyzed in serum only in nine patients, in CSF only in four patients, and in paired serum and CSF samples in the remaining 24 patients. Of the patients tested with paired samples, four had antibodies detected in only CSF (four anti‐NMDAR), and five had antibodies detected in only serum (three anti‐LGI1, one anti‐CASPR2, one anti‐Ma2/Ta) (Table 1). Two patients had multiple neuronal antibodies: one 53‐year‐old woman with Morvan's syndrome had both anti‐CASPR2 and anti‐LGI1 antibodies; one 75‐year‐old woman with limbic encephalitis and an ovarian tumor had anti‐NMDAR, anti‐AMPA, anti‐Hu, and anti‐neurexin‐3α in serum and anti‐NMDAR, anti‐Hu, and anti‐CV2 in CSF.

#### 3.2.2 Magnetic resonance tomography

**FIGURE 2:**
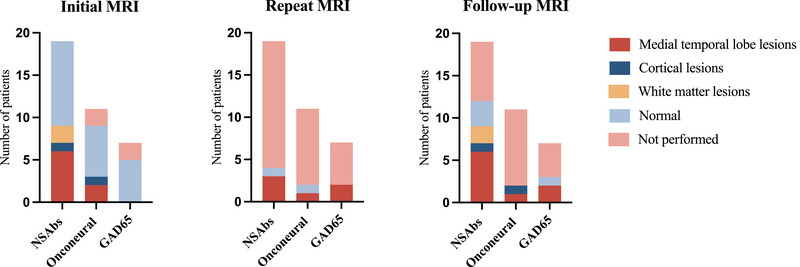
Results of brain magnetic resonance imaging (MRI); Initial MRI was performed at the onset of neurological symptoms and before the start of immunotherapy; repeat MRI was performed after the initial MRI but before the start of immunotherapy; follow‐up MRI was performed after the start of immunotherapy. NSAbs, neuronal surface antibodies.

An initial brain MRI scan after the onset of neurological symptoms before immunotherapy was performed in 32 patients, of whom 11 (30%) had an abnormal finding (Figure 2). Eight patients underwent a repeat brain MRI scan before the start of immunotherapy, six of whom had an abnormal finding (four of these six had a normal initial brain MRI). All 19 patients with NSAbs underwent initial brain MRI, six (32%) of whom had increased T2 signal in one or both medial temporal lobes. Two patients had white matter lesions suggestive of demyelinating disease on initial brain MRI (both had anti‐NMDAR encephalitis), and they were subsequently diagnosed with multiple sclerosis (MS) and radiologically isolated syndrome (RIS), respectively. Follow‐up MRI scans after the start of immunotherapy were obtained in 17 of 37 patients (median 6 months after the start of immunotherapy, range 0–27 months). Only one patient (6%) had a complete regression of abnormalities on follow‐up scan after initiation of immunotherapy, whereas the other patients had progression of abnormalities in one case (6%), unchanged findings in nine cases (53%), partial regression in four cases (24%), and new findings in two cases (12%).

#### 3.2.3 Cerebrospinal fluid analyses

**FIGURE 3:**
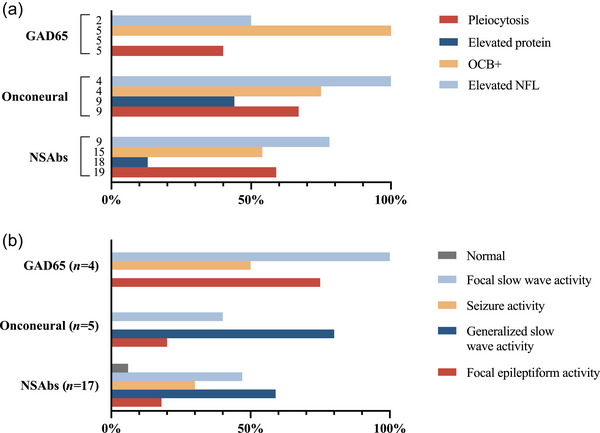
(a) Cerebrospinal fluid (CSF) analysis after the start of neurological symptoms and before the start of immunotherapy for each antibody subgroup. Columns show the percentage of patients with pathological results for each CSF analysis. Numbers to the left of columns indicate the number of patients tested for each analysis. NFL, neurofilament light chain; OCB+, oligoclonal bands. (b) Electroencephalography (EEG) findings after the start of neurological symptoms before the start of immunotherapy. Columns show the percentage of patients with each EEG pattern. Patients with more than one EEG pattern are represented in multiple columns. Numbers in parentheses indicate the number of patients analyzed in each antibody subgroup. NSAbs, neuronal surface antibodies.

Lumbar puncture with CSF analysis was performed in 31 patients after the onset of neurological symptoms and before the start of immunotherapy (Figure 3a). The NSAbs subgroup had pleocytosis, elevated protein, positive oligoclonal bands, or a combination of the three in 12 of 17 (71%) cases. If elevated neurofilament light chain (NFL) levels were included, 15 of 17 (88%) patients had abnormal findings. The remaining two patients with a normal CSF analysis had anti‐LGI1 and anti‐CASPR2 encephalitis. In the onconeural subgroup, 8 of 9 (89%) patients had either pleocytosis, elevated protein, positive oligoclonal bands, or a combination of the three, and 5 of 5 (100%) patients with anti‐GAD65 disease had the same. NFL levels before the start of immunotherapy were highest in the onconeural subgroup (median 35,200 ng/L, range 14,900–55,800, 100% above age‐adjusted cut‐off value), followed by the NSAbs subgroup (median 4000 ng/L, range 750–9320, 78% above age‐adjusted cut‐off value) and the anti‐GAD65 subgroup (median 1445 ng/L, range 1440–1450, 50% above age‐adjusted cut‐off value).

#### 3.2.4 Electroencephalography

Twenty‐six patients underwent EEG recording (Figure 3b) after the start of neurological symptoms and before the start of immunotherapy (when given). Half of the patients with anti‐GAD65 antibodies, 30% of patients with NSAbs, and none of the patients with onconeural antibodies had electroencephalographic seizures recorded. There were various ictal patterns, and many electrographic seizures had no clinical correlation. All patients, except for one case with anti‐CASPR2 encephalitis, had an abnormal EEG recording.

#### 3.2.5 Computed tomography scan

An abdominal and thoracic CT scan was performed in 27 patients after the onset of neurological symptoms (16 with NSAbs, 10 with onconeural antibodies, and one with anti‐GAD65 antibodies). A third of scans (9/27) detected a tumor, of which only one was known beforehand.

#### 3.2.6 Positron emission tomography

Whole‐body 18‐fluorodeoxyglucose positron emission tomography (FDG‐PET) was performed in 13 patients (five with NSAbs, five with onconeural antibodies, and three with anti‐GAD65 antibodies). A tumor was detected in 6 of 13 patients (46%), of whom four had a previous abdominal and thoracic CT scan without signs of malignancy. Seven patients underwent brain FDG‐PET, six of which had pathological findings. Only one patient had abnormalities on the brain FDG‐PET, yet a normal brain MRI (a case of anti‐CASPR2 encephalitis).

### 3.3 Timeline

**FIGURE 4:**
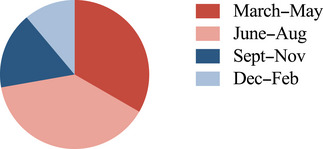
The month for disease onset for patients with neuronal surface antibodies (NSAbs) (*n* = 14) and anti‐GAD65 (*n* = 4).

The onset of symptoms showed a seasonal variation in patients with NSAbs (*n* = 14) and anti‐GAD65 antibodies (*n* = 4), with most patients (72%) falling ill in the spring or summer (Figure 4). Five patients with NSAbs met the criteria for definite PNS and were excluded from the analysis, and three patients with anti‐GAD65 had missing data.

At symptom onset, most patients first visited the Department of Internal Medicine (Table 3). All but one patient required in‐patient care (97%), eight (22%) of whom required intensive care. Two patients were admitted to compulsory in‐patient psychiatric care: one suffering from anti‐NMDAR encephalitis and one from anti‐GAD65 encephalitis. There was no significant difference in the number of days between first contact to the start of immunotherapy between patients with NSAbs (median: 35, range: 1–924 days), onconeural antibodies (median: 30, range: 5–156 days), and anti‐GAD65 antibodies (median: 213, range: 5–1754 days; *p* = .26).

**TABLE 3:** Timeline of healthcare contacts.

	NSABS (*n* = 19)	ONCONEURAL (*n* = 11)	GAD65 (*n* = 7)	ALL (*n* = 37)
FIRST CONTACT^a^				
NEUROLOGY DEPARTMENT, *n* (%)	4 (21)	2 (18)	2 (33)^b^	8 (22)
PSYCHIATRIC DEPARTMENT, *n* (%)	0 (0)	0 (0)	0 (0)^b^	0 (0)
INTERNAL MEDICINE DEPARTMENT, *n* (%)	9 (47)	5 (45)	2 (33)^b^	16 (43)
GENERAL PRACTITIONER, *n* (%)	5 (26)	2 (18)	2 (33)^b^	9 (24)
OTHER, *n* (%)	1 (5)	2 (18)	0 (0)	3 (8)
HOSPITALIZED, *n* (%)	19 (100)	11 (100)	5 (83)^b^	37 (97)^b^
DAYS HOSPITALIZED, median (range)	44 (8–94)^c^	35 (12–90)	34 (11–43)^b^	35 (6–94)^d^
ICU, *n* (%)	6 (32)	2 (18)	0 (0)	8 (22)
DAYS AT ICU, median (range)	17 (2–25)	25 (5–45)	0^b^	17 (2–45)
INVOLUNTARY COMMITMENT^e^, *n* (%)	1(5)	0 (0)	1 (14)	2 (5)
DAYS TO DIAGNOSIS^f^, median (range)	35 (1–929)	12.5 (1–78)^b^	249 (14–3535)^c^	29 (1–3535)^d^
IMMUNOTHERAPY				
DAYS ANTIBODY RESULT TO THERAPY^g^ (MEDIAN, RANGE)	3 (0–48)	7 (0–78)^h^	49 (11–59)	5 (0–78)
THERAPY STARTED BEFORE ANTIBODY^i^, *n* (%)	3 (16)	0 (0)	4 (57)	7 (19)
DAYS FIRST CONTACT TO THERAPY, MEDIAN (RANGE)	35 (1–924)^c^	30 (5–156)^c^	213 (5–1754)^c^	35 (1–1754)^k^
RELAPSE, *n* (%)	1 (5)	0 (0)	0^b^(0)	1 (3)^α^
DECEASED, *n* (%)	4 (21)	7 (64)	1 (14)^ε^	12 (32)
MONTHS TO DEATH, median (range)	12 (0.5–60)	4.5 (1.5–34)	22^l^	6 (0.5–60)
FOLLOW‐UP IN MONTHS, median (range)	33 (1–63)	17 (2–47)	34 (22–73)	33 (1–73)

Abbreviation: NSAbs, neuronal surface antibodies.

^a^ The department where the patient was first seen.

^b^ Missing data on one patient.

^c^ Missing data on two patients.

^d^ Missing data on three patients.

^e^ Involuntary commitment to a psychiatric ward.

^f^ Days from first contact to detection of auto‐antibody.

^g^ days from detection of auto‐antibody to start of immunotherapy.

^h^ Two patients did not receive immunotherapy.

^i^ Immunotherapy was initiated before confirmation of diagnosis with the auto‐antibody test result.

^j^ Days from first contact to start of immunotherapy.

^k^ Missing data on six patients.

^l^ Suicide.

To explore if the type of onset would affect the time until treatment interventions were initiated, we assigned the patients with NSAbs into one of two categories: acute onset (anti‐NMDAR encephalitis, anti‐GABA‐B encephalitis) or insidious onset (anti‐LGI1 encephalitis, anti‐CASPR2 encephalitis, anti‐IgLON5 encephalopathy). Patients with acute onset received treatment with immunotherapy earlier (median: 16 days; range 1–49) than patients with an insidious onset (median: 134 days; range 9–929; *p* = .0016) from the first contact.

After confirmation of the diagnosis with a positive antibody test, most patients with NSAbs received immunotherapy quickly (median 3 days, range 0–48), and 16% had already started immunotherapy before the results of the antibody test came back. More than half of the patients with anti‐GAD65 antibodies had received immunotherapy when the antibody test results returned.

### 3.4 Treatment

#### 3.4.1 Symptomatic treatment

Anti‐epileptic drugs (AEDs) were used in 62% of patients, with multiple AEDs used particularly in the GAD65 subgroup (Table 4). The most frequently used AED was levetiracetam, the first choice in 17 out of 23 patients. Psychiatric medications (antidepressants or neuroleptics) were used in more than half of the patients (57%). The most frequently used neuroleptic was haloperidol, followed by olanzapine.

**TABLE 4:** Treatments received by patients during the disease course.

	NSABS (*n* = 19)	ONCONEURAL (*n* = 11)	GAD65 (*n* = 7)	ALL (*n* = 37)
AED, *n* (%)	14 (74)	4 (36)	5 (71)	23 (62)
AED ≥ 2	7 (37)	3 (27)	4 (57)	14 (38)
AED ≥ 5	2 (11)	0 (0)	4 (57)	6 (16)
PSYCHIATRIC MEDS, *n* (%)	11 (58)	5 (45)	4 (57)	21 (57)
ANTIDEPRESSANTS^a^	3 (16)	3 (27)	4 (57)	11 (30)
NEUROLEPTICS^b^	8 (42)	2 (18)	0 (0)	10 (27)
IMMUNOTHERAPY, *n* (%)	19 (100)	9 (82)	7 (100)	35 (95)
STEROIDS IV	17 (90)	6 (55)	3 (43)	26 (70)
IVIG	13 (68)	7 (64)	6 (86)	26 (70)
STEROIDS ORAL	6 (32)	3 (27)	4 (57)	13 (35)
PLASMAPHERESIS	1 (5)	2 (18)	1 (14)	4 (11)
RITUXIMAB	12 (63)	3 (27)	7 (100)	22 (60)
CYCLOPHOSPHAMIDE	1 (5)	1 (9)	0 (0)	2 (5)
TOCILIZUMAB	1 (5)	0 (0)	0 (0)	1 (3)

*Note*: Rows 2 and 3 show patients who received two or more different AEDs or five or more different AEDs, respectively.

Abbreviation: AED, antiepileptic drugs.

^a^ Antidepressants = fluoxetine, mirtazapine, citalopram, ecitalopram, paroxetine, venlafaxine, sertraline, duloxetine, and clomipramine.

^b^ Neuroleptics = olanzapine, quetiapine, haloperidol, zuclopenthixol, risperidone, clozapine, and aripiprazole.

#### 3.4.2 Immunotherapy

Most patients received immunotherapy (95%; Table 4). The exceptions were one patient with anti‐Ma2/Ta encephalitis with spontaneous recovery and one patient with paraneoplastic anti‐Hu encephalitis, who was considered to be in a palliative stage and subsequently died. Rituximab was prescribed to 22 patients (60%), of whom 10 received at least one repeated dose 6 months after the initial dose. Of these 10 patients, five received rituximab every 6 months until the last follow‐up; two cases of anti‐NMDAR encephalitis due to a coexisting diagnosis of MS and RIS, respectively; one case of anti‐GAD65‐associated Stiff person syndrome disorder; one case of anti‐GAD65‐associated cerebellopathy; and one case of anti‐Yo paraneoplastic cerebellar degeneration. Only one patient with anti‐LGI1 encephalitis (who had received i.v. steroids, intravenous immunoglobulins, and a single dose of rituximab) had a relapse 2 years later, necessitating renewed treatment.

#### 3.4.3 Oncological treatment

Of the 16 patients with cancer, two had received cancer treatment (surgery and chemotherapy) before the first symptoms of PNS started (two women with ovarian carcinoma and paraneoplastic cerebellar degeneration). The other 14 patients with cancer were diagnosed after the neurological symptoms started, and 10 patients subsequently received cancer treatment (surgery *n* = 3, chemotherapy *n* = 10, radiotherapy *n* = 5). None of the patients received cancer immunotherapy.

## 4 DISCUSSION

In this retrospective case series of 37 patients with AE and PNS in Sweden, we described the clinical characteristics and disease course based on the autoantibodies associated with these disorders. Notably, while our findings generally align with earlier studies from different global regions regarding sex distribution, clinical presentation, and ancillary test outcomes, certain differences stood out (Lancaster et al., 2010; Liem et al., 2023; Madlener et al., 2023; Titulaer et al., 2013; van Sonderen et al., 2016; van Sonderen et al., 2016; Vogrig et al., 2020).

One pronounced divergence was the age distribution, particularly amongst our anti‐NMDAR encephalitis patients, who were notably older than those in prior studies (Bastiaansen et al., 2022; Dalmau et al., 2008; Gong et al., 2021; Irani et al., 2010; Titulaer et al., 2013), even those from neighboring countries (Nissen et al., 2021). The spectrum of detected autoantibodies in our case series was similar to that of some previous studies (Iorio et al., 2020; Liem et al., 2023; Vogrig et al., 2020). In contrast, other studies might suggest that anti‐NMDAR antibodies should have been the most prevalent antibody subtype, as our case series did not exclude children (Deng et al., 2019; Hébert et al., 2020; Swayne et al., 2021; Wright et al., 2015). Our cases were identified in our previous epidemiological study of AE and PNS in Sweden (Kosek et al., 2023), in which the incidence rate of anti‐NMDAR encephalitis was low compared with other studies (Bastiaansen et al., 2022; Nissen et al., 2021). A few potential cases of anti‐NMDAR encephalitis were excluded from our epidemiological study due to lack of consent. Still, the age distribution of excluded patients did not differ from that of included patients and thus would not have changed the findings of this study even if they were included. While earlier studies indicate that AE patients frequently have initial consultations with psychiatric departments due to the commonality of psychiatric symptoms at onset (Dalmau et al., 2008; Kayser et al., 2013), none in our cohort did. Instead, the majority first consulted internal medicine. This discrepancy might reflect Sweden's healthcare structure, but it also signals a need for greater awareness amongst Swedish healthcare practitioners about AE, particularly in younger patients.

The diagnosis of AE can be challenging, especially for the subgroups with insidious onset such as anti‐LGI1 encephalitis, anti‐CASPR2 encephalitis, and anti‐IgLON5 encephalopathy. A notable diagnostic delay has previously been reported (Ariño et al., 2016; Gaig et al., 2017; Grüter et al., 2022; Muñiz‐Castrillo et al., 2021; Rodriguez et al., 2021; van Sonderen, Ariño et al., 2016; van Sonderen, Thijs et al., 2016). In our study, there was a significant difference in the time to treatment between patients with anti‐NMDAR and anti‐GABA‐B encephalitis compared with the more insidious anti‐LGI1 encephalitis, anti‐CASPR2 encephalitis, and anti‐IgLON5 encephalopathy (more than eight times longer). Although MRI and CSF analysis (if NFL was included) were abnormal in more than half of the cases of anti‐LGI1 encephalitis, these tests were often performed in close proximity to antibody testing and near the start of immunotherapy. The diagnostic delay could potentially have been avoided with increased awareness of the clinical presentation. One example is the presence of early faciobrachial dystonic seizures (FBDS), which were seen in the majority of our anti‐LGI1 encephalitis cases and considered pathognomonic for anti‐LGI1 encephalitis (Irani et al., 2011). Recognition of the clinical presentation in different anti‐GAD65‐associated disorders can also be challenging. In our study, patients with anti‐GAD65‐associated disorders had the longest time to treatment initiation, in line with previous studies showing a long diagnostic delay (Madlener et al., 2023). Overall, our case series suggests that increased awareness of the clinical presentation of these disorders should be promoted in order to avoid diagnostic delays in the future.

Metrics like the duration of hospital stays and ICU admissions can offer insights into disease severity and healthcare efficiency (Rotter et al., 2010). These measures may be subject to cultural and structural differences between different units in the same geographical region (rural hospitals vs. tertiary centers) and between different geographical regions. Previous studies of hospitalized patients with AE have demonstrated that these patients require long hospital stays (Harutyunyan et al., 2017; Hasbun et al., 2017; Swayne et al., 2021), longer than patients with infectious meningitis and encephalitis (Hasbun et al., 2017). Our study results align with these previous findings regarding length of hospital stay. The proportion of patients admitted to the ICU was lower in our study than in previously described cohorts (Titulaer et al., 2013), but the length of stay was longer (Harutyunyan et al., 2017; Mittal et al., 2016), perhaps reflecting different traditions in ICU admission rather than disease severity. Long hospitalizations likely reflect the severity of the disease as Sweden overall has a low average length of stay in hospitals compared with other OECD countries (OECD, 2023).

A considerable seasonal variation in the onset of AE has been reported, with patients falling ill in the warm seasons (Adang et al., 2014; Bastiaansen et al., 2022; Lai et al., 2021). Similar seasonal patterns of disease onset have been reported in multiple sclerosis (MS) and neuromyelitis optica spectrum disorders (Akaishi et al., 2020; Harding et al., 2017). In our study, patients with NSAbs and anti‐GAD65 antibodies without cancer had onset of symptoms predominantly in the Swedish spring or summer months. Seasonal variation in the relapse rate of MS has been demonstrated to be latitude‐dependent, with variation increasing by the distance from the equator (Spelman et al., 2014). Associations with environmental factors such as low sunlight exposure or infections associated with a latitudinal gradient have been suggested. Whether the seasonal variation noted in the onset of AE is also latitude‐dependent is yet unknown. Viruses, such as Herpes simplex virus (HSV) and Japanese encephalitis virus, are known to trigger AE (Hacohen et al., 2014; Linnoila et al., 2016; Ma et al., 2017). In our cohort, one patient with anti‐NMDAR encephalitis had prodromal HSV type 1 encephalitis; however, in the other patients, there were no documented viral infections. Further studies regarding seasonal or latitudinal variations in AE and their association with environmental factors might elucidate the underlying autoimmune mechanisms of the disease.

This study has several limitations. A retrospective review of medical records is liable to missing or biased data. For example, we could not present information about clinical outcomes as physicians rarely noted patients’ functional outcomes in their notes in a manner that allowed for grading. Nor could we provide the rationale behind the choice of therapy, as this was rarely described. The small sample size in this study limits the interpretation of the data and the generalizability of the findings. Nevertheless, these insights remain valuable given the rarity of AE and PNS and the potential for geographical variations.

In conclusion, our Swedish cohort showcased similarities with global AE and PNS data but with marked differences in age distribution for anti‐NMDAR encephalitis cases. There was a seasonal variation in the onset of non‐tumor‐related AE, and AE with a more insidious onset was associated with delayed treatment initiation. These findings emphasize the need for heightened clinical awareness and vigilance toward these disorders among European clinicians.

## AUTHOR CONTRIBUTIONS

**Sonja Kosek**: Investigation; writing—original draft; visualization; conceptualization; formal analysis. **Joachim Burman**: Conceptualization; funding acquisition; writing—review and editing; supervision. **Anna Rostedt Punga**: Conceptualization; funding acquisition; writing—review and editing; supervision.

## CONFLICT OF INTEREST STATEMENT

The authors declare no conflicts of interest.

### PEER REVIEW

The peer review history for this article is available at https://publons.com/publon/10.1002/brb3.3534.

## Data Availability

The datasets analyzed during the current study are not publicly available due to the GDPR legislation, but are available from the corresponding author upon reasonable request.
